# Enhancing accuracy of detecting left atrial dilatation on CT pulmonary angiography

**DOI:** 10.1016/j.ejro.2025.100696

**Published:** 2025-10-15

**Authors:** Louis Tapper, Samer Alabed, Ahmed Maiter, Andrzej Lejawka, Mahan Salehi, Krit Dwivedi, Pankaj Garg, David G. Kiely, Peter Metherall, Rob J. van der Geest, Kavita Karunasaagarar, Michael Sharkey, Andrew J. Swift

**Affiliations:** a Sheffield Teaching Hospitals, Sheffield, UK; b NIHR Sheffield Biomedical Research Centre, UK; c Division of Clinical Medicine, School of Medicine & Population Health, University of Sheffield, UK; d University of East Anglia, Norwich Medical School, Norwich, UK; e Norfolk and Norwich University Hospitals NHS Foundation Trust, Norwich, UK; f Leiden University Medical Centre, Leiden, Netherlands

## Abstract

**Introduction:**

Left atrial (LA) dilatation predicts several cardiovascular disorders. Identifying LA dilatation on computed tomography pulmonary angiography (CTPA) could aid diagnosis of cardiovascular disease. This study assessed an artificial intelligence (AI) segmentation model’s performance at detecting LA dilatation on CTPA.

**Methods:**

Patients with suspected pulmonary hypertension (PH) who underwent CTPA and cardiac MRI (CMR) were retrospectively identified from a single centre registry. The LA was segmented by an AI tool for CTPA and a validated AI tool for CMR. LA volume measurements were categorised for LA dilatation based on existing threshold values. The expert radiologist's reports of the CTPA studies were also categorised for LA dilatation. Automated CTPA LA volumes and corresponding radiologist reports were compared against the reference standard of CMR.

**Results:**

451 patients were included (mean age 64 ± 13 years, 62.5 % female, 85.8 % white). Automated LA volume measurements on CTPA showed strong positive correlation with those on CMR (ρ = 0.92, p < 0.001) with minimal bias on Bland-Altman analysis (-4 mL, 95 %CI −39 to +31 mL). Automated LA measurements on CTPA showed higher agreement with those on CMR (κ = 0.80) than the radiologist reports (κ = 0.62). Automated LA measurements on CTPA showed higher accuracy metrics (sensitivity 81.0 %, specificity 96.8 %, positive predictive value (PPV) 88.5 %, negative predictive value (NPV) 94.4 %) than the radiologist reports (sensitivity 66.7 %, specificity 93.1 %, PPV 74.5 %, NPV 90.2 %).

**Conclusion:**

Deep learning increases the accuracy of LA volume measurements on non-ECG gated CTPA, improving radiologist performance in detecting LA dilatation.

## 1 Introduction

Although CMR is considered the gold standard for non-invasive assessment of cardiac chamber volumes, [1] there is growing interest in the ability to measure chamber volumes on computed tomography (CT) images. Not only is CT more widely available than CMR, but it is also faster to acquire and often better tolerated by patients. CMR provides excellent soft tissue contrast, but CT provides superior spatial resolution, potentially enabling more precise volumetric measurements. However, a limitation for accurate volumetry in many thoracic CT studies is the lack of ECG-gating. Thoracoabdominal CT studies are performed for a variety of indications and almost always capture at least part of the heart, offering opportunities for opportunistic screening of cardiac disease. CTPA is one of the most common cross-sectional imaging studies, captures the whole heart within its field of view, and includes contrast within the cardiac chambers. Dilatation of the LA on imaging is a recognised predictor for several cardiovascular diseases, including atrial fibrillation, systemic embolism and cardiac failure.[2], [3] Accurate quantification of LA chamber volume on CTPA could enable opportunistic disease feature finding for LA dilatation, prompting further investigation for subclinical cardiovascular disease or the initiation of risk-modifying interventions such as prophylactic anticoagulation.

Measuring the volume of a cardiac chamber on CMR, CTPA and other imaging requires *segmentation*: the delineation of the chamber’s anatomical boundaries on the images. Traditionally, segmentation has been performed manually by cardiac imaging experts, a task that is laborious, repetitive and prone to both intra- and interobserver variability. Even for experts, accurate assessment and delineation of the LA and other cardiac chambers can be challenging. Chamber morphology and volume vary during the cardiac cycle and are influenced by a plethora of physiological and pathological factors. Cardiac motion can cause artefact and limit spatial resolution, and is a particular challenge in patients with arrhythmia. The techniques for quantifying LA volume differ between imaging modalities and observers, with no universally accepted threshold value to define dilatation.

In recent years, there has been a marked rise in the number of AI-based tools for the automated segmentation of cardiac structures on CMR,[4.], [5] which have aimed to improve the efficiency and reliability of evaluating chamber volumes and other features on CMR.[6], [7] Few studies have attempted automated segmentation on non-ECG-gated CT studies such as CTPA. [6]

We have previously reported the development of a deep learning model for automated segmentation of the heart and great vessels on CTPA. [8] This study aimed to assess the performance of this CTPA AI segmentation model for automated segmentation and volume measurement of the LA on CTPA.

## 2 Methods

**Fig. 1:**
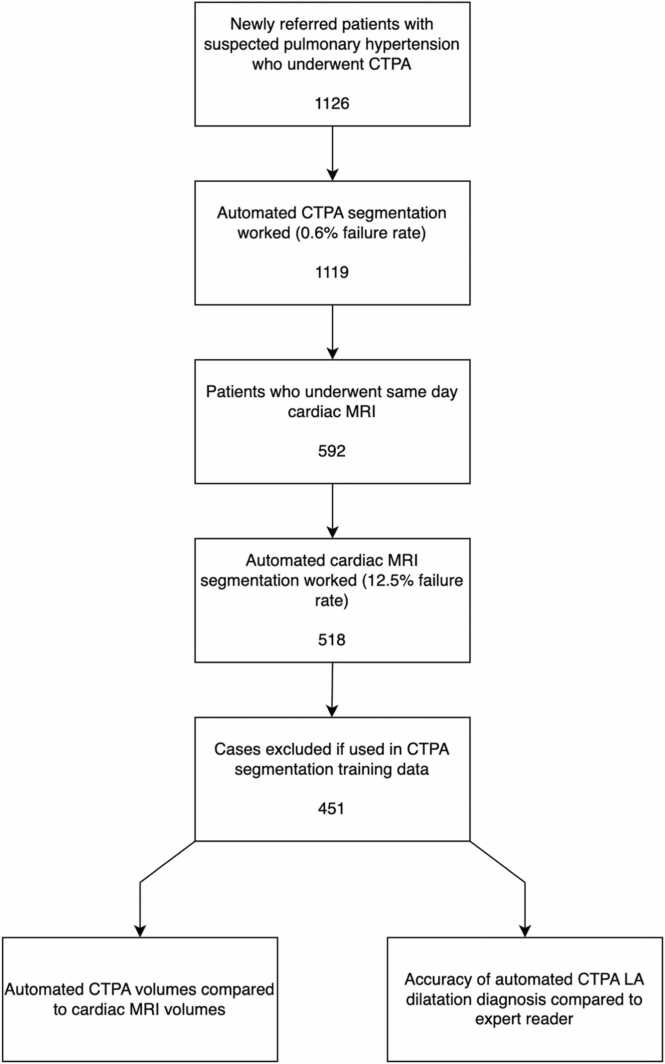
Study flow diagram.

**Fig. 2:**
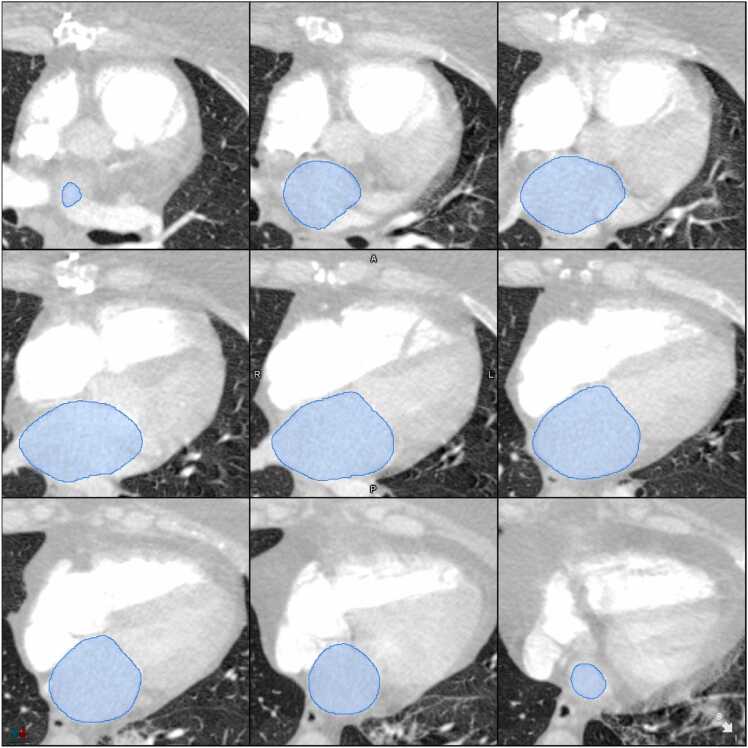
CT with segmentation. Example case: CTPA LA volume reported as normal by radiologist. CTPA AI LA volume: 125 mL. CMR AI LA volume: 106 mL. RHC wedge pressure: 29 mmHg.

This retrospective study was approved by a local ethics committee and institutional review board (c06/Q2308/8). The need for dedicated patient consent was waived. The study flow is indicated in Fig. 1, Fig. 2.

### 2.1 Patient selection

Consecutive patients referred with newly suspected PH were retrospectively identified from the “Assessing the Spectrum of Pulmonary hypertension Identified at a REferral centre” (ASPIRE) registry. [9] This is a database of patients evaluated for suspected pulmonary vascular disease at Sheffield Teaching Hospitals NHS Foundation Trust. Adult patients (aged ≥ 18 years) were eligible for inclusion if they had undergone both non-ECG-gated CTPA and CMR on the same day between 2011 and 2019. Patients were excluded if they did not undergo both CTPA and right heart catheterisation (RHC) within 48 h as this would suggest that they had known chronic PH. Pseudo-anonymised demographic information, CTPA and CMR images with corresponding radiologist reports, and final clinical diagnosis were retrieved from the registry for each eligible patient. Protocols for CTPA and CMR image acquisition are provided in the Supplementary Materials.

### 2.2 Image analysis

The CTPA AI model performed automated segmentation of the LA on the CTPA images. Development and prior testing of the model have been reported previously [8] but are described here in brief. A deep convolutional neural network architecture based on nn-UNet was trained to undertake segmentation of nine structures including the cardiac chambers, ventricular myocardium and pulmonary arteries and thoracic aorta. CTPA studies from 200 patients assessed for suspected PH in Sheffield were used for development (80/20/100 training/validation/internal testing subsets) with CTPA studies from 20 patients used for external testing. This external testing dataset was from hospitals across England and Wales. Manual contouring by consultant cardiac radiologists was used as the reference standard. The Dice similarity coefficients for segmentation of the LA were 0.91 (95 %CI 0.90–0.92) and 0.87 (95 %CI 0.84–0.90) in the internal and external test sets for the final model. Failure analysis was performed for 1333 patients with pulmonary vascular disease, with failure of LA segmentation observed in only 0.6 % of cases. Automated segmentation of the LA on CTPA images by the AI model was performed using three-dimensional volumetric data, with direct calculation of LA volume. Please see Sharkey MJ et al. [8] for further details including failure analysis, comparison between automated and manual CTPA LA segmentation and a segmentation pipeline. No external validation cases have been included in the cohort for this study.

Automated segmentation of the LA on CMR images was performed by the software MASS (version 2020 EXP; Leiden University Medical Center, the Netherlands), which has been validated. [7] Automated segmentation was performed on two-chamber and four-chamber views. The biplanar method was used to determine maximal LA volume, with the atrial length and area from both the two-chamber and four-chamber views averaged and the LA volume calculated using the following formula: 0.85 x averaged atrial area / averaged atrial length. [10]

The LA appendage was excluded from segmentation on both CTPA and CMR. Patients for which CTPA or CMR segmentation failed (e.g. in due to poor contrast opacification or excessive artefact) were excluded *post hoc*. [8]

### 2.3 Radiological reportshttp

All CTPA studies had been reported as part of routine clinical practice by specialist cardiothoracic radiologists with at least 10 years experience. The reports were retrospectively reviewed by two radiology residents (LT and AL) and categorised as either normal LA volume or LA dilatation. In cases where LA dilatation was not explicitly reported, normal LA size was assumed. The information extracted from the reports would likely be based on a visual assessment, possibly with the aid of diameter or area measurement. This reporting process was not standardized and reflected routine clinical practice.

### 2.4 Statistical analysis

The LA volumes derived from automated LA segmentation on CTPA were compared to the reference standard of LA volumes derived from automated LA segmentation on CMR. Firstly, the automated quantitative measurements were compared directly using intra-class correlation coefficient and Bland-Altman analysis. Secondly, the volumes were categorised as either normal or consistent with LA dilatation; this was performed using threshold values of > 100 mL in females and > 112 mL in males based on a previous meta-analysis. [10] The agreement of these categorised outcomes was then assessed using Cohen’s kappa statistic (κ) which was interpreted according to previously reported thresholds: 0.00–0.20 none, 0.21–0.39 minimal, 0.40–0.59 weak, 0.60–0.79 moderate, 0.80–0.90 strong, > 0.90 almost perfect. [11]

Additionally, the diagnostic accuracy for detection of LA dilatation of the automated CTPA segmentation and that of the CTPA clinical reports were each assessed using contingency tables with CMR as the reference standard. The conventional metrics of sensitivity, specificity, PPV, NPV and area under the curve (AUC) were derived.

Statistical analysis was performed using R version 4.2.2. The significance threshold was set at p = 0.05.

## 3 Results

### 3.1 Characteristics of included patients

451 consecutive patients were included (mean age 64 ± 13 years, 62.5 % female, 85.8 % white). The demographics and clinical characteristics are presented in Table 1, Table 2. LA dilatation was identified by the reference standard of automated LA segmentation on CMR in 23.2 % of cases.

**Table 1:** na.

**Demographics**	
Age, years ± SD	64 ± 13
Female, % (n)	62.5 (282/451)
Ethnicity % (n)	
White	85.8 (399/451)
Asian	4.4 (20/451)
Black	2.0 (9/451)
Any other ethnic group	1.1 (5/451)
Not documented	4.0 (18/451)

**Table 2:** na.

**Diagnoses % (n)**	
Pulmonary arterial hypertension	19.5 (88/451)
Left heart disease	18.1 (82/451)
Lung disease	15.7 (71/451)
Chronic thromboembolic disease	31.5 (142/451)
Unclear / multifactorial	2.0 (9/451)
Not pulmonary hypertension	13.1 (59/451)

### 3.2 Performance of automated left atrial segmentation on CTPA

The failure rate for LA segmentation on CTPA was 0.6 %. Failures were predominantly associated with low or no contrast in the chamber. Other causes included pericardial effusions and chamber dilatation. [9]

### 3.3 Comparison of LA volume measurements derived from automated segmentation on CTPA and CMR

**Fig. 3:**
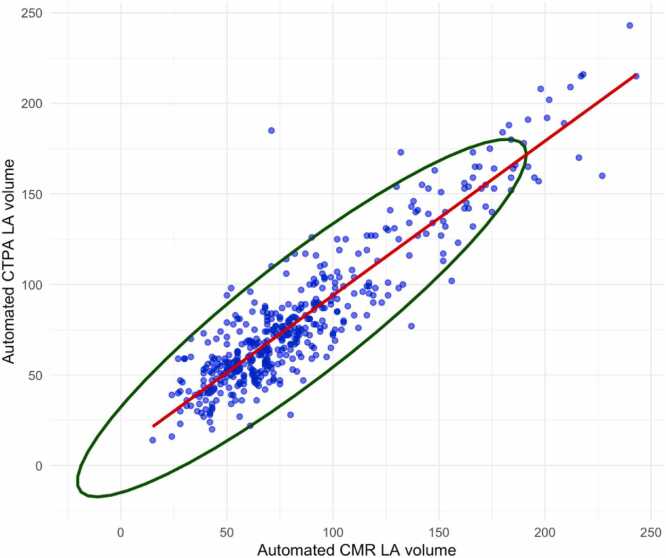
Shows a scatterplot of CT LA volume against CMR LA volume measures.

**Fig. 4:**
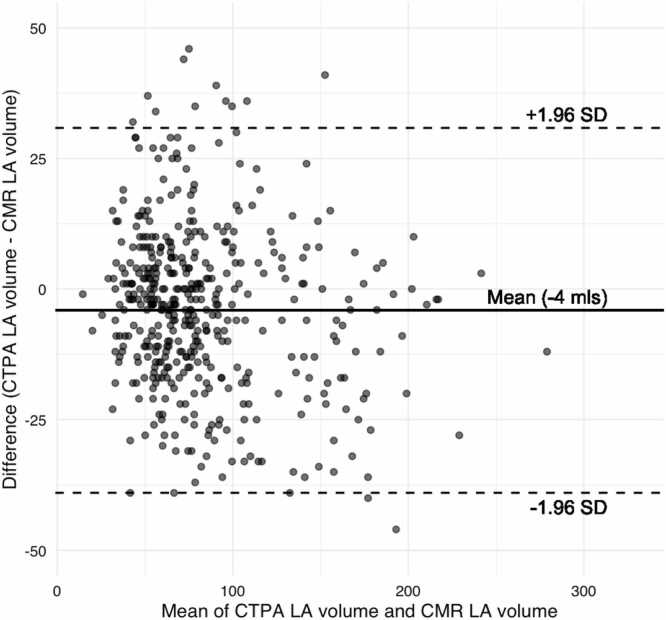
Bland Altman plot of LA volumes derived from CTPA against CMR.

Automated segmentation yielded mean LA volumes of 82 ± 42 mL on CTPA and 86 ± 46 mL on CMR. The LA volumes on CTPA demonstrated a strong and statistically significant positive correlation (ρ = 0.92, p < 0.001) with those on CMR (Fig. 3). Bland-Altman analysis indicated a mean difference of only −4 mL (95 %CI −39 to +31 mL) between LA volumes on CTPA and CMR (Fig. 4).

Strong agreement was identified between the categorised LA volumes derived automatically from CTPA and CMR (κ = 0.80, 95 % CI= 0.73–0.86, p < 0.001). Moderate agreement was found between the categorised radiologist CTPA reports and categorised LA volumes derived automatically from CMR (κ = 0.62, 95 % CI= 0.53–0.7, p < 0.001).

### 3.4 Comparison of categorised LA volumes derived from automated segmentation on CTPA and CMR

Diagnostic accuracy metrics for automated detection of LA dilatation was as follows: sensitivity 81.0 % (95 % CI: 72.1–88.0 %), specificity 96.8 % (95 % CI: 94.4–98.4 %), PPV 88.5 % (95 % CI: 80.4–94.1 %), NPV 94.4 % (95 % CI: 91.4–96.5 %) (Table 3).

**Table 3:** Diagnostic accuracy, sensitivity, specificity, PPV and NPV for CT AI prediction of LA dilatation.

	CMR dilated	CMR non-dilated	
CTPA AI dilated	85	11	PPV: 88.5 %
CTPA AI non-dilated	20	335	NPV: 94.4 %
	Sensitivity: 81.0 %	Specificity: 96.8 %	

### 3.5 Comparison of categorised CTPA reports and categorised LA volumes derived from automated segmentation on CMR

In comparison the sensitivity for expert reader detection of LA dilatation was 66.7 % (95 % CI: 56.8–75.6 %) and specificity 93.1 % (95 % CI: 89.9–95.5 %). PPV: 74.5 % (95 % CI: 64.4–82.9 %). NPV: 90.2 % (95 % CI: 86.6–93.1 %) (Table 4).

**Table 4:** Diagnostic accuracy, sensitivity, specificity, PPV and NPV for Radiologist prediction of LA dilatation.

	CMR dilated	CMR non-dilated	
CTPA reader dilated	70	24	PPV: 74.5 %
CTPA reader non-dilated	35	322	NPV: 90.2 %
	Sensitivity: 66.7 %	Specificity: 93.1 %	

### 3.6 ROC analysis

**Fig. 5:**
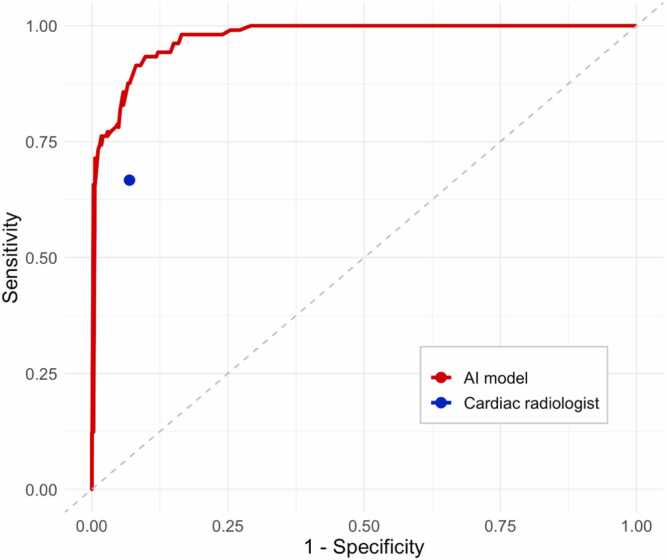
Receiver operating characteristic curve showing automatic CTPA prediction of LA dilatation versus expert Radiologist prediction. AUC 0.975 (95 %CI 0.963–0.987, p < 0.001). Threshold of 89.5 mL yields the maximum Youden’s Index of 0.835 with sensitivity 0.933 and specificity 0.902.

The ROC curve for the LA volumes derived by automated segmentation on CTPA is shown in Fig. 5. The AUC for the automated segmentation was 0.975 (95 % CI: 0.963–0.987, p < 0.001). The sensitivity and specificity of the categorised CTPA reports was plotted as a single point for comparison as there was no equivalent continuous variable.

## 4 Discussion

This study assessed the performance of a deep learning model for automated LA segmentation, volume quantification and detection of dilatation on CTPA. Patients who underwent both CTPA and CMR on the same day were retrospectively identified from the ASPIRE registry. The LA was segmented automatically on CTPA by the deep learning model, and on CMR by previously validated software, yielding chamber volumes which were categorised for dilatation according to existing thresholds. There was a strong positive correlation between the automated volume measurements on CTPA and CMR, with minimal bias on Bland-Altman analysis. When compared against those on CMR, the automated volume measurements on CTPA showed strong agreement by Cohen’s κ, greater than that of the categorised radiologist reports. Our study demonstrates the ability of a deep learning model for segmentation of cardiac structures on CTPA to yield accurate measurements of LA volume that can be used to reliably determine the presence of LA dilatation. More broadly, our study highlights the feasibility of AI-based tools to perform accurate segmentation and meaningful assessment of cardiac chambers on non-ECG-gated CT examinations - indicating a potential avenue of opportunistic screening for chamber dilatation.

The categorised LA volumes obtained from automated segmentation on CTPA yielded universally higher values across all diagnostic accuracy metrics (i.e. sensitivity, specificity, PPV and NPV) than the categorised radiologist reports when compared against the reference standard of CMR. ROC analysis showed a high AUC for automated segmentation. The results suggest that the CTPA AI segmentation model could outperform expert readers at the detection of LA dilatation, and use of the model in reporting might assist evaluation of the LA and possibly other cardiac chambers during routine CTPA reporting.

The patients included from the ASPIRE registry all underwent both CTPA and CMR within 48 h. This is a major strength of our study, as it minimises the risk of confounding physiological factors (such as hydration status) on chamber volume. As aforementioned, the threshold values used to classify chamber volumes as dilated or non-dilated can vary. We used threshold values from a published meta-analysis [10], improving the external validity of the study design. Threshold values can be changed as clinical guidance evolves, and implementing such changes is likely to be more straightforward and reliable in an automated pipeline than for human reporters.

We recognise the limitations of our study. Patients were included from a single centre registry. All patients had been referred for evaluation of suspected PH and therefore had a higher prevalence of LA dilatation compared to the general population. [12] However, this study primarily aimed to demonstrate the feasibility of a segmentation model for accurately quantifying LA volume and detecting LA dilatation on CTPA - further research will be required to determine whether the performance demonstrated generalises to the broader patient population, such as those patients undergoing CTPA for suspected acute pulmonary embolism. The increased prevalence of LA dilatation in our study cohort could potentially contribute to overestimation of the CTPA AI segmentation model’s performance, and again further testing in a more unselected patient cohort is important for verifying generalisability. Cases in which automated segmentation on either CTPA or CMR were excluded, although these were few in number and are unlikely to have had significant impact on the results. Automated segmentation of the LA on CMR by a validated AI-based tool was used to provide the reference standard. Although CMR is the gold standard investigation for non-invasive measurement of chamber volumes, there is no universally agreed technique for doing so; the biplane method is most common and has been used here. Lastly, the performance of the expert readers may have been underestimated due to the use of historical reports rather than prospective blinded evaluation. In routine practice the expert readers would routinely evaluate the LA and comment if it was abnormal. We postulate a general radiologist would comment even less frequently.

The study findings support further evaluation of the CTPA AI segmentation model. Volume measurements and classification of dilatation could also be evaluated for the other cardiac chambers. Diagnostic accuracy metrics of the model outperformed those of the radiologist reports, and prospective evaluation of radiologist reporting with and without the model would help to determine potential benefits to clinical practice. The model could also be tested alongside other tools for cardiac segmentation on CT in a comparative study.

## 5 Conclusion

This study assessed the performance of an AI deep learning model for automated LA volumetry on CTPA. The model had good agreement with the current gold standard which is CMR. It yielded accurate LA volumes, and outperformed radiologist reports at the detection of LA dilatation. We have demonstrated the feasibility of using automated segmentation on non-ECG-gated CT studies to measure LA volume.

## Funding statement

This study/research is funded by the National Institute for Health and Care Research (NIHR) Sheffield Biomedical Research Centre (NIHR203321). The views expressed are those of the author(s) and not necessarily those of the NIHR or the Department of Health and Social Care. This work was supported by an NIHR AI Award, AI_AWARD01706. This research was funded in whole, or in part, by the 10.13039/100004440Wellcome Trust 223521/Z/21/Z.

## Figures and tables

Demographic and clinical characteristic tables

## Ethical Statement

All procedures were performed in compliance with relevant laws and institutional guidelines.

This retrospective study was approved by a local ethics committee and institutional review board (c06/Q2308/8).

## CRediT authorship contribution statement

**Andrzej Lejawka:** Writing – original draft, Data curation. **Mahan Salehi:** Writing – review & editing, Data curation. **Krit Dwivedi:** Writing – review & editing. **Pankaj Garg:** Writing – review & editing. **David G Kiely:** Writing – review & editing, Resources. **Peter Metherall:** Writing – review & editing, Software, Resources. **van der Geest Rob J:** Writing – review & editing, Software, Resources. **Kavita Karunasaagarar:** Writing – review & editing, Conceptualization. **Michael Sharkey:** Writing – review & editing, Software, Resources, Funding acquisition, Formal analysis, Data curation, Conceptualization. **Louis Tapper:** Writing – review & editing, Writing – original draft, Visualization, Methodology, Investigation, Formal analysis, Data curation. **Andrew J Swift:** Writing – review & editing, Visualization, Supervision, Software, Resources, Methodology, Funding acquisition, Data curation, Conceptualization. **Samer Alabed:** Writing – review & editing, Writing – original draft, Visualization, Supervision, Software, Methodology, Formal analysis, Conceptualization. **Ahmed Maiter:** Writing – review & editing, Data curation.

## Declaration of Competing Interest

The authors declare the following financial interests/personal relationships which may be considered as potential competing interests: Samer Alabed reports financial support was provided by NIHR BRC Sheffield. Andrew J Swift reports financial support was provided by NIHR BRC Sheffield. Krit Dwivedi reports financial support was provided by NIHR BRC Sheffield. Michael Sharkey reports financial support was provided by NIHR BRC Sheffield. Samer Alabed reports a relationship with Executive commitee British Society of Cardiovascular Imaging that includes: board membership. Ahmed Maiter reports a relationship with British Institute of Radiology that includes: travel reimbursement. Krit Dwivedi reports a relationship with Pulmovant that includes: consulting or advisory. David Kiely reports a relationship with Speaker fees & honoraria from Acceleron, Altavant, Apollo, Ferrer, Gossamer-Bio, GSK, Janssen, Liquidia, Merck and United Therapeutics. His department has received grants from MRC, BHF, NIHR, Wellcome Trust, Ferrer, GSK and Janssen. that includes: funding grants and speaking and lecture fees. Michael Sharkey reports a relationship with National Institute for Health and Care Research that includes: funding grants. Ahmed Maiter reports a relationship with Royal College of Radiologists that includes: travel reimbursement. Clinical advisor for Pie Medical Imaging and Medis Medical Imaging, and he consults for Anteris and Edwards Lifesciences- Pankaj Garg

This was supported by an NIHR AI Award, AI_AWARD01706- Michael Sharkey.

This research was funded in whole, or in part, by the Wellcome Trust 223521/Z/21/Z- Michael Sharkey. If there are other authors, they declare that they have no known competing financial interests or personal relationships that could have appeared to influence the work reported in this paper.
