# Supplementary material for: Enhancing accuracy of detecting left atrial dilatation on CT pulmonary angiography
Source: Eur J Radiol Open. 2025 Oct 15;15:100696. doi: 10.1016/j.ejro.2025.100696 (PMC12552999; doi:10.1016/j.ejro.2025.100696)
Supplement: Supplementary file 1 — Supplementary material [file mmc1.docx]

# **Supplementary material**

## **CTPA protocol**

CTPA was performed on a 64 detector-row CT GE system (Light-Speed; General Electric Medical Systems, Milwaukee, WI) between 2011 and 2017 and on a 320 detector-row Canon CT system (Aquilion ONE/ViSION edition; Canon Medical Systems Corporation, Otawara, Japan) between 2017 and 2019. GE acquisition parameters were as follows: 120 kV, 100 mA with auto dose reduction, pitch 1, rotation time 0.5 s, field of view (FOV) 400 × 400mm and slice thickness 0.625 mm. Canon acquisition parameters were as follows: kV 120, modulated mA, pitch (standard pitch factor 0.813 and helical pitch 65) rotation time 0.275, FOV 500 L and slice thickness 0.3 mm. Intravenous iodinated contrast agent (Omnipaque 300, GE Healthcare, United States) was administered with a dose of 60 ml at a rate of 5 ml/s. Bolus tracking was used with a region of interest over the pulmonary trunk with a manual trigger. Contiguous slices were acquired during an inspiratory breath hold.

## **MRI protocol**

CMR was performed with 1.5 Tesla MRI systems from GE (Signa HDx, General Electrics Healthcare). Short-axis cine images were acquired using a cardiac-gated multislice balanced steady-state free precession sequence (20 frames per cardiac cycle, section thickness 10mm, 0mm inter-section gap, field of view 480mm, acquisition matrix 256 × 200, flip angle 60°, BW 125 KHz/pixel, TR/TE 3.7/1.6ms). A stack of images in the short-axis plane was acquired, fully covering both ventricles from base to apex. End-systole was considered to be the smallest cavity area. End-diastole was defined as the first cine phase of the R-wave triggered acquisition or largest volume. Patients were supine with a surface coil and with retrospective ECG gating.
